# PET Imaging in Vulvar Cancer: A Literature Review of the Current Evidence and Clinical Applications

**DOI:** 10.3390/cancers18142308

**Published:** 2026-07-17

**Authors:** Rayan Gazawi, Hanin Lataifeh, Abdulla Alzibdeh, Marwah Abdulrahman, Akram Al-Ibraheem, Fawzi Abuhijla

**Affiliations:** 1 Department of Radiation Oncology, King Hussein Cancer Center, Amman 11941, Jordan; 2 Department of Internal Medicine, Unity Health—White County Medical Center, Searcy, AR 72143, USA; 3 Department of Nuclear Medicine, King Hussein Cancer Center, Amman 11941, Jordan

**Keywords:** vulvar cancer, PET scan, theranostic imaging, molecular imaging, adaptive radiotherapy

## Abstract

Vulvar cancer is a rare gynecologic malignancy with a bimodal age distribution, predominantly affecting older women while demonstrating increasing HPV-related incidence in younger populations. Accurate staging is essential for individualized treatment strategies to optimize outcomes while minimizing morbidity. Positron Emission Tomography (PET) has emerged as an important imaging modality in oncology; however, its precise role in vulvar cancer requires critical evaluation given the limited prospective evidence. This review focuses on the role of ^18^F-FDG PET/CT in vulvar cancer based on the literature published to date. Current evidence suggests that PET/CT provides high sensitivity for primary tumor detection and is particularly valuable for nodal and distant staging in locally advanced disease, where it may significantly influence management. However, its limited spatial resolution precludes detection of micrometastases, and it cannot replace sentinel lymph node biopsy for groin staging. PET/CT also contributes to radiotherapy planning through improved target delineation and demonstrates utility in detecting recurrence, although false positives remain a limitation. MRI remains superior for local staging, supporting a complementary multimodality imaging approach. Emerging tracers, including Fibroblast Activation Protein Inhibitor (FAPI), along with artificial intelligence-based radiomics, represent promising but still investigational directions. In conclusion, ^18^F-FDG PET/CT is a useful adjunct in vulvar cancer, especially for advanced disease, but its interpretation should be integrated within a multimodal framework due to limited supporting evidence.

## Simple Summary

This literature review explains why better imaging is needed for vulvar cancer, a rare disease where knowing the exact extent of the tumor and lymph node spread changes treatment and outcomes. The authors summarize current evidence on whole-body metabolic imaging using FDG PET/CT, describe how it helps detect larger nodal and distant disease and assist radiotherapy planning, and explain its limits, especially that it misses very small lymph node deposits and cannot replace sentinel node biopsy. They also outline promising new tracers and machine learning tools that might improve detection and treatment planning in the future. By clarifying where PET/CT is useful and where more research is needed, the paper aims to guide clinicians and researchers toward studies that could refine staging and improve personalized care for patients with vulvar cancer.

## 1. Introduction

Gynecological malignancies (GM) comprise a heterogeneous group of cancers with distinct diagnostic pathways, management strategies, and prognostic outcomes. Vulvar cancer is a rare GM: U.S. Surveillance, Epidemiology, and End Results (SEER) data report an age-adjusted incidence of 2.6 per 100,000 women per year, a lifetime risk of approximately 0.3%, and a 5-year relative survival of 69.7%. The median age at diagnosis is 70 years, and 63.0% of incident cases occur in women aged 65 years or older (26.7% aged 65–74, 23.2% aged 75–84, and 13.1% aged over 84); nevertheless, the disease follows a bimodal pathway, with human papillomavirus (HPV)-associated tumors contributing to disease in younger women [1,2]. Imaging is critical for staging and treatment planning: magnetic resonance imaging (MRI) is superior for assessing local tumor extent [3], while Positron Emission Tomography with 2-deoxy-2-[fluorine-18]fluoro-D-glucose integrated with computed tomography (^18^F-FDG PET/CT) enables whole-body metabolic evaluation of lymph nodes and distant metastases, emerging as a potentially valuable tool for preoperative staging, treatment response assessment, and recurrence detection. However, evidence supporting its routine use in vulvar cancer remains limited, and it has yet to be incorporated into standardized clinical practice [4]. In this literature review, we examine the clinical utility of ^18^F-FDG PET/CT, either alone or in combination with MRI or CT, in the diagnostic evaluation and management of vulvar cancer, with particular emphasis on its role in guiding radiotherapy.

Because this is a literature review without an institutional or local patient cohort, it does not present local outcomes. Instead, it synthesizes the existing literature, delineates situations in which PET/CT is selectively indicated, and identifies questions for which future local validation would provide clinically useful evidence.

## 2. Methods

A review of the literature was conducted to identify relevant studies on the role of PET in vulvar cancer. The search encompassed the PubMed/Scopus database for articles published between January 1999 and December 2025. The search strategy employed MeSH terms, including (“Vulvar Neoplasms”[Mesh]) AND (“Positron Emission Tomography”[Mesh] OR “Fluorodeoxyglucose F18”[Mesh]) AND (“Radiotherapy”[Mesh] OR “Radiotherapy, Adjuvant”[Mesh]) AND (FAPI OR “fibroblast activation protein inhibitor” OR “FAPI PET” OR “^68^Ga-FAPI”). The search was limited to peer-reviewed original articles, systematic reviews, and meta-analyses published in English. The reference lists of included articles were also screened to identify additional relevant studies. This work constitutes a narrative review; accordingly, it does not employ a PRISMA-based study-selection flowchart, predefined inclusion and exclusion criteria, or a formal risk-of-bias assessment, and the included studies were not formally graded. Because vulvar cancer is rare and dedicated PET evidence is limited, the search was deliberately broad, and supporting evidence was drawn from related gynecologic and squamous cell malignancies (e.g., cervical, breast, and head and neck cancer); throughout this review, such extrapolated evidence is presented as supportive and hypothesis-generating and is explicitly distinguished from vulvar-specific data.

## 3. Principles of ^18^F-FDG PET/CT Imaging in Vulvar Cancer

Since its introduction in the 1990s, ^18^F-FDG PET has emerged as a reliable diagnostic modality that provides functional information about tissue metabolism, complementing the anatomic detail obtained from conventional imaging techniques such as CT and MRI. PET imaging is based on the detection of pairs of high-energy photons generated during positron–electron annihilation events. A positron (e^+^) from a tracer annihilates with an electron (e^−^), producing two 511 keV photons emitted in opposite directions. Their coincident detection defines a line of response (LOR), and many such events are used to reconstruct a three-dimensional tracer distribution [5,6]. The most commonly used positron-emitting isotope in clinical practice is fluorine (^18^F), which is incorporated into a glucose analog known as ^18^F-fluoro-D-glucose (^18^F-FDG), which follows the glucose transport pathway from plasma into cells. However, unlike glucose, FDG is not further metabolized and becomes trapped intracellularly. This property allows PET imaging to reflect tissue glucose metabolism. In malignant cells, increased glycolysis is associated with overexpression of glucose transporters (GLUT-1 and GLUT-3) and elevated mitochondrial hexokinase activity. When PET is combined with anatomic imaging modalities such as CT or MRI, it enables precise localization of metabolically active tissues, thereby aiding in diagnosis, treatment planning, and monitoring response to therapy [7]. FDG PET/CT interpretation for nodal metastasis in vulvar cancer varies across studies and lacks standardized diagnostic criteria. In clinical practice, assessment is primarily based on qualitative visual analysis, supported by semiquantitative parameters. Lymph nodes are generally considered suspicious when they demonstrate focal FDG uptake greater than background activity and not attributable to physiological or inflammatory causes, particularly when corresponding to nodal stations on CT. Semiquantitative evaluation using SUVmax may aid interpretation; however, no universally accepted cutoff exists, and reported thresholds in the literature are heterogeneous and study-dependent. In Batog et al.’s study, the mean SUVmax for metastatic (true-positive) nodes was 7 (range, 1.6–30.0), whereas for histologically negative (false-positive) nodes it was 2.18 (range, 1.9–3.1) [8].

## 4. Overview of the Staging System in Vulvar Cancer

Diagnosis of vulvar cancer is confirmed through punch or incisional biopsy of the lesion. Staging of GM is performed according to the International Federation of Gynecology and Obstetrics (FIGO) criteria and is initially guided by imaging studies, with final staging determined by sentinel lymph node biopsy. Under the FIGO 2021 classification, vulvar cancer is divided into four stages. As demonstrated by Table 1, Stage I disease is confined to the vulva or perineum and is subdivided into IA and IB. Stage II is characterized by a tumor of any size extending to the lower third of the urethra, vagina, or anus without lymph node involvement. Stage III disease includes tumors of any size extending to the upper portions of perineal structures or involving non-fixed, non-ulcerated lymph nodes. This stage is further subdivided into IIIA, IIIB, IIIC. Stage IV disease represents tumor involvement of bone, fixed or ulcerated lymph nodes, or distant metastasis [9,10]. Assessment of nodal involvement in vulvar cancer is of paramount importance due to its strong prognostic implications.

For the criteria unique to FIGO staging, the role of PET/CT is well defined as compared with its universal applicability to all FIGO stages. For stage I tumors, there is no need to replace PET/CT with clinical evaluation, MRI in cases with local extension, and sentinel lymph node biopsy (SLNB), except in cases in which evaluation of the groin is difficult, the main tumor is large or deeply involved, and SLNB is not possible. For stage II tumors, PET/CT is valuable in situations where there is a possibility of invasion of the urethra, vagina, or anal canal, and the conventional imaging cannot demonstrate the nodal or distant involvement preoperatively or preradiotherapy. PET/CT is valuable in stage III tumors to define the extent of involvement of inguinal/femoral and pelvic nodes, unexpected distant metastases, to perform biopsies of suspicious nodes, and to design radiotherapy fields. In the case of stage IV or recurrent tumors, PET/CT is basically used as a whole-body staging/re-staging imaging tool to decide on the feasibility of salvaging treatment or to opt for a systemic approach [11,12,13,14].

## 5. Role of Imaging Management Decision in Vulvar Cancer

Imaging plays a central role in the initial evaluation and staging of vulvar and other GMs. A multimodal imaging approach is commonly used, incorporating pelvic and abdominal ultrasound (US), CT, MRI, and ^18^F-FDG PET/CT. Current guidelines (e.g., ESGO 2023, NCCN) recommend MRI as the primary modality for local staging of vulvar cancer due to its high soft-tissue resolution. In contrast, ^18^F-FDG PET/CT is primarily reserved for cases with suspected metastatic involvement of inguinofemoral or pelvic lymph nodes, equivocal findings on MRI or CT, or advanced disease (≥T3), as it provides functional assessment that complements anatomical imaging and can guide biopsy or treatment planning when distant or nodal metastases are a concern [11,12,13].

MRI is preferred as the adjunctive modality when local invasion must be defined: in stages I–II, it is used to measure tumor size and depth and to assess extension into the urethra, vagina, or anus; in stages III–IV, it better delineates upper urethral or vaginal, bladder, rectal, pelvic sidewall, and bone involvement and supports radiotherapy planning. PET/CT complements MRI primarily by assessing inguinofemoral or pelvic nodes and distant disease rather than replacing MRI for local T staging [3,11,12,13].

Risk-adapted imaging selection is increasingly emphasized in pelvic malignancies, as modality performance may vary according to patient- and tumor-related factors such as body habitus, tumor location, prior treatment, and suspected extent of disease. In vulvar cancer, MRI remains the preferred modality for local soft-tissue assessment; ultrasound may be useful for superficial inguinofemoral nodal evaluation and image-guided biopsy, whereas FDG PET/CT is most valuable in clinically suspicious nodal disease, locally advanced tumors, suspected distant metastasis, equivocal conventional imaging, recurrence, or radiotherapy planning. Although comparative evidence in vulvar cancer remains limited, similar risk-adapted frameworks in rectal cancer have shown that MRI and ultrasound performance can differ according to BMI, tumor location, and neoadjuvant treatment status, supporting a personalized rather than uniform approach to imaging selection [15].

PET imaging enables whole-body evaluation in a single, minimally invasive examination. However, its intrinsic spatial resolution (approximately 4–7 mm) limits sensitivity for small lesions, particularly in the subcentimeter range, where detectability depends on lesion-to-background contrast. Hybrid imaging with CT (PET/CT) provides attenuation correction and precise anatomical localization, thereby improving diagnostic accuracy. In selected settings, PET/MRI may offer additional soft-tissue characterization. This combined approach has significantly enhanced the role of PET in gynecologic malignancies, although challenges such as intense urinary FDG activity and adjacent pelvic structures may still affect lesion conspicuity [16,17]. Overall diagnostic performance of PET has been reported with a sensitivity of 80%, specificity of 90%, positive predictive value (PPV) of 80%, and negative predictive value (NPV) of 90% [18]. Weissinger et al. revealed that patients who underwent whole body FDG PET/MRI had a high specificity in nodal staging (97.2%) but revealed limited sensitivity (66.7%) due to micrometastases less than 2 mm, and combining both SLN mapping and FDG PET/MRI increased sensitivity to (77.8%); which acts as complementary for specifically detecting para-aortic lymph node involvement and possible distant organ metastases [19]. A systematic review and meta-analysis that was done by Triumbari et al. revealed that a negative preoperative PET/CT scan can almost exclude pelvic metastases in early-stage vulvar cancer in patients who are unable to undergo sentinel lymph node biopsy [14].

### Clinical Algorithm for the Use of ^18^F-FDG PET/CT in Vulvar Cancer

**Figure 1:**
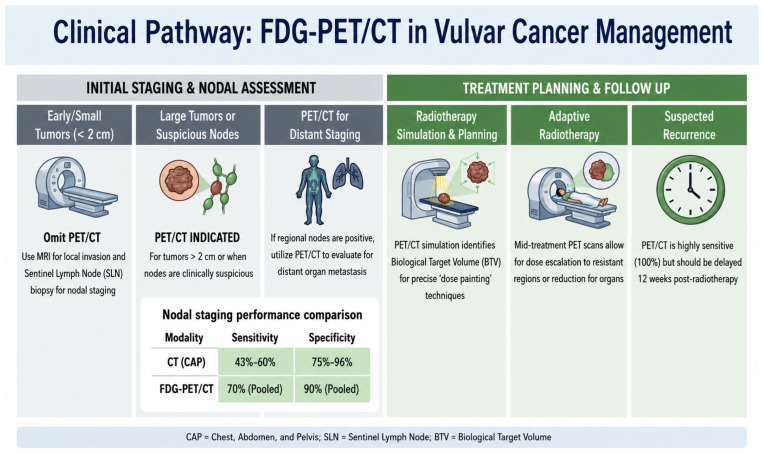
Clinical algorithm for the use of FDG PET/CT in vulvar cancer.

The clinical use of ^18^F-FDG PET/CT in vulvar cancer should be guided by disease stage and clinical context. In early-stage disease (≤T2, clinically node-negative), sentinel lymph node biopsy remains the standard of care, and PET/CT is not routinely indicated due to limited sensitivity for micrometastases. In contrast, in locally advanced disease (≥T3), or when nodal involvement is suspected or equivocal on conventional imaging, PET/CT provides valuable whole-body assessment and may influence treatment planning. PET/CT is particularly useful in detecting distant metastases and in evaluating suspected recurrence. Importantly, PET/CT findings should always be interpreted in conjunction with histopathology and anatomical imaging, especially in cases of discordant results. The algorithm presented in Figure 1 is not a validated decision rule but an author-proposed synthesis intended to summarize current practice. It was derived by integrating the staging-based recommendations of the ESGO 2023 and NCCN guidelines with the diagnostic-performance evidence discussed in this review—in particular the high negative predictive value of PET/CT and the meta-analytic data supporting its selective use in advanced or equivocal disease [11,12,14]—and is therefore intended as a pragmatic framework rather than a prescriptive standard, to be applied alongside multidisciplinary judgment.

Thus, this review suggests that the use of PET/CT should not be performed on a regular basis for patients with non-metastatic vulvar cancer. The added value of PET/CT before confirmed metastasis is limited to certain high-risk or equivocal situations: large, deep, or locally advanced tumors; clinically or sonographically suspicious inguinal nodes; equivocal MRI or CT results; when SLNB is contraindicated because of previous surgery, lymphangiographic changes, patient’s physical condition, or technical limitations; and in cases where the delineation of radiotherapy targets is required. For small, unifocal, clinically node-negative tumors eligible for SLNB, PET/CT is suggested to be regarded as an option rather than a standard of care [11,12,13,14].

If there are no correlative MRI or CT findings for the PET-positive abnormality, there is no need for escalation of treatment. It can be helpful to perform a multidisciplinary review of PET/CT, pelvic MRI, CT component, clinical evaluation, infection/inflammation history, and availability of the lesion to biopsy. When the result would change the FIGO stage, the method of operation, the radiotherapy fields, or the systemic therapy, image-guided biopsy or fine needle aspiration is recommended to be performed whenever possible. If the biopsy cannot be safely done or is technically impossible, short-term follow-up by imaging or assessment of treatment response is preferred to the treatment of the PET-positive signal itself [11,12,13].

Consistent implementation requires multidisciplinary training for nuclear medicine physicians, radiologists, radiation oncologists, gynecologic oncologists, surgeons, and pathologists. A practical competency pathway should standardize patient preparation, acquisition and reconstruction parameters, semiquantitative measurements, recognition of physiologic and inflammatory uptake, joint review of PET/CT with pelvic MRI or diagnostic CT, and audit of imaging interpretations against biopsy or surgical pathology. Management-changing positive findings should undergo multidisciplinary review and targeted tissue confirmation whenever feasible, reducing the risk of unnecessary inguinofemoral lymphadenectomy or inappropriate enlargement of radiotherapy fields [11,12,13,20,21].

Patients should also be counseled that equivocal or false-positive uptake may cause anxiety and lead to repeat imaging or biopsy. PET/CT adds fasting and uptake time, a 20–30 min acquisition, ionizing-radiation exposure, cost, travel, and access burdens; these factors should be weighed against the likelihood that the result will change management [22,23].

Organ preservation should be framed as an indirect, stage-dependent benefit rather than a stand-alone indication for PET/CT. Reliable identification of macroscopic spread may support less extensive surgery or more conformal radiotherapy, potentially preserving vulvar and perineal structures and avoiding morbidity from complete inguinofemoral lymphadenectomy. However, PET/CT cannot by itself justify omission of sentinel lymph node biopsy or otherwise indicated surgery because micrometastases may be missed; histopathologic confirmation and stage-based management remain required [11,12,13,14,24,25,26,27].

## 6. Role of PET Imaging in Radiotherapy Contouring, Planning, and Adaptive Radiotherapy

**Figure 2:**
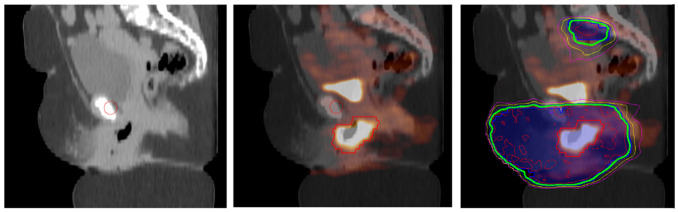
Left-to-right sagittal views: CT simulation image, CT simulation fused with PET FDG scan, and radiotherapy isodoses distribution to primary disease and pelvic nodes.

Radiation therapy planning is a complex, multidisciplinary process that relies heavily on imaging to ensure accurate delivery of therapeutic radiation doses while minimizing exposure to surrounding normal tissues [28,29]. The metabolic information provided by ^18^F-FDG PET/CT allows for identification of the anatomical gross tumor volume (GTV) as well as the biological target volume (BTV), which reflects areas of increased tumor activity [30,31,32]. Integration of PET data into radiotherapy planning enables the concept of dose painting, in which radiation doses are delivered in a non-uniform manner based on functional tumor characteristics. Dose painting may involve either dose escalation or dose redistribution. The latter strategy aims to increase radiation delivery to biologically aggressive or radioresistant sub-volumes while reducing exposure to surrounding normal tissues, thereby optimizing therapeutic efficacy while limiting toxicity. The simplest method of incorporating PET information into radiotherapy planning is visual or cognitive fusion of PET with CT or MRI scans, as shown in Figure 2. In this approach, clinicians manually compare images to guide target volume contouring and make adjustments for anatomic or spatial discrepancies. However, this method is limited by intra- and inter-observer variability, which can reduce precision. A more accurate approach is PET/CT simulation, in which automated image fusion is performed during simulation. Although more precise, this technique is associated with increased cost and resource utilization [33,34].

Curative radiotherapy aims to deliver tumoricidal doses to malignant tissue while accounting for potential microscopic disease extension. Manual segmentation based on visual assessment remains the most commonly used method for target volume delineation; however, this approach is highly operator-dependent and requires significant expertise regarding tracer properties and uptake mechanisms. More recently, automated and semi-automated segmentation techniques have been developed, using SUV thresholds or cutoff values to define contour boundaries. Despite their potential advantages, these techniques may under-detect small tumors and can be inaccurate when target lesions are adjacent to organs with high physiologic tracer uptake, such as the bladder [33]. This is attributed to the partial volume effect (PVE), where metabolic signals from small volumes smear into adjacent non-active pixels, resulting in significantly underestimated SUV and subsequent contour contraction [35].

Postoperative radiotherapy is recommended to be initiated as early as feasible, with an average start time of 104 days following surgical excision. Indications for adjuvant radiotherapy include positive surgical margins when further resection is not possible. In cases of close but negative margins accompanied by extensive lymphovascular space invasion, perineural invasion, or lymph node involvement, radiotherapy may be considered on an individualized basis to reduce local recurrence. Sentinel lymph node metastases measuring less than or equal to 2 mm, as well as isolated tumor cells, may be managed with postoperative radiotherapy as an alternative to inguinofemoral lymphadenectomy to reduce long-term morbidity. Following inguinofemoral lymphadenectomy, radiotherapy is recommended for patients with more than one metastatic lymph node and/or extracapsular spread, with or without concurrent radiosensitizing chemotherapy. For unresectable disease or cases requiring extensive surgery, primary chemoradiotherapy is the preferred treatment approach. Treatment response is typically assessed approximately 12 weeks after completion of therapy through clinical examination, imaging, or repeat biopsy when residual disease is suspected. In recurrent or metastatic vulvar cancer, no standardized treatment strategy currently exists. Palliative chemotherapy has demonstrated response rates ranging from 14% to 40% [36,37], and emerging evidence suggests a role for targeted immunotherapy. PET imaging may assist in measuring metabolically active gross tumor volume, providing insight into tumor burden and aiding radiation therapy planning [4].

PET also plays an important role in adaptive radiotherapy planning, where treatment strategies are modified based on early response to therapy. Follow-up PET imaging is typically performed within the first several weeks of treatment, allowing for radiation dose escalation to treatment-resistant regions or dose reduction to minimize exposure to organs at risk. This approach has demonstrated benefit in esophageal, lymphoma, and lung cancers [38,39,40,41]. In the study by Shenker et al., 20 patients treated with PET/CT-guided adaptive radiotherapy experienced statistically significant reductions in radiation dose to the bladder, bowel, and rectum, with median reductions of 1.1 Gy (*p* < 0.001), 1.0 Gy (*p* < 0.001), and 0.66 Gy (*p* = 0.006), respectively. At two years, local control, disease-free survival, and overall survival rates were 63%, 43%, and 68%, respectively [42].

## 7. Diagnostic Utility of PET Versus CT and MRI

### 7.1. Local Staging

^18^F-FDG PET/CT demonstrates high sensitivity for detecting primary vulvar tumors, particularly in squamous cell carcinoma, with some studies reporting up to 100% sensitivity for primary lesion detection [43,44]. However, it has also been reported to have a limited benefit in local staging due to inadequate soft-tissue resolution [4,8]. MRI is therefore considered a widely preferred imaging modality for evaluating the extent of local invasion in primary vulvar cancer due to its superior soft-tissue contrast [45]. This has been supported by Sohaib et al., who reported correct preoperative staging in 70% of patients with primary vulvar SCC using pelvic MRI [46]. Additionally, in a retrospective study by Kataoka et al., adding contrast improved lesion detection and accuracy from 75% to 85% in 20 patients [47].

### 7.2. Lymph Node Staging

The evaluation of lymph node involvement remains a critical component of vulvar cancer staging. CT of the chest, abdomen, and pelvis (CT CAP) has been investigated for nodal assessment, with studies reporting modest sensitivity but consistently high specificity. Across several retrospective and prospective studies, CT CAP sensitivity for detecting nodal metastases ranged from 43% to 60%, while specificity ranged from 75% to 96% [48,49,50,51]. PPV ranged from 58% to 62%, and NPV ranged from 73% to 76%, indicating that CT CAP can be used to exclude metastasis if findings are negative.

The role of ^18^F-FDG PET/CT in nodal staging has been extensively explored in both prospective and retrospective studies. Collectively, studies demonstrated considerable variability in reported sensitivity of groin and pelvic lymph node metastases, as demonstrated in Table 2, ranging from approximately 50% to 100% [18,36,43,52,53,54,55,56,57,58]. Several studies reported high sensitivity (79–100%) and advocated for the use of PET/CT in preoperative nodal assessment [24,36,43,52,54,58]. In contrast, other investigations observed lower sensitivity (50–80%) and emphasized the need for cautious interpretation of positive PET/CT findings, recommending histologic confirmation when feasible [18,36,53]. Despite this heterogeneity, specificity was generally high across studies, often exceeding 78%, and NPV was consistently strong, ranging from 88 to 96% in larger cohorts. These findings suggest that a negative PET/CT scan can strongly predict the absence of nodal metastases, which safely identifies patients who are candidates for sentinel lymph node biopsy [24,57].

This general sensitivity rate of 50–100% across studies may be due to many sources of heterogeneity in the methodology and clinical aspects of the studies. Older PET or PET/CT systems were used, as well as different reconstruction techniques. Also, there was variability in uptake time, and there were variations in CT and MRI correlation approaches. There was also significant patient selection heterogeneity, varying from early stages of cancer patients, where they are clinically node negative, to more advanced cases or restaging, where nodal involvement is relatively common. The unit of analysis used included patient level, groin level, or nodal level, and the reference standard varied from SLNB or lymphadenectomy to imaging or clinical follow-up. Other sources of heterogeneity include small-volume lesion size, biology, inflammation, and SUVmax cut-off [14,18,24,36,52,53,54,55,56,57,58].

Across the vulvar cancer studies summarized in Table 2, negative predictive value (NPV) was generally high (77–96%), but specificity ranged from 66% to 100%, corresponding to a false-positive rate (1—specificity) of 0–34%. Thus, a negative scan can be reassuring for macroscopic nodal disease, whereas a positive scan has a materially higher risk of false positivity and should not prompt lymphadenectomy or radiotherapy escalation without anatomical correlation and, when feasible, histologic confirmation [14,18,24,36,52,53,54,55,56,57,58].

### 7.3. Distant Metastasis

Given the relative rarity of vulvar cancer and its low incidence of distant spread, data regarding imaging for detection of pelvic lymph nodes and distant metastases remain limited. In a study by Robertson et al., FDG PET identified distant metastasis in 10 of 54 patients, compared with 5 detected by conventional CT, with PET/CT revealing additional metastatic lesions not visualized on CT alone [55]. Conversely, Lin et al. reported that CT-CAP or MRI demonstrated greater overall efficacy than FDG-PET/CT for detecting pelvic nodal or distant metastases, although PET/CT identified an additional true-positive pancreatic metastasis at the expense of a higher false-positive rate [52]. Clinical implications of these findings remain unclear, as discordant findings should be resolved in favor of histopathology and properly reviewed by a multidisciplinary team for proper management, with PET/CT serving as an adjunct rather than a determinant of surgical decision-making.

## 8. PET Imaging in Follow-Up and Detection of Recurrence

Long-term surveillance is a critical component of vulvar cancer management, as recurrence remains a significant contributor to morbidity and mortality. Studies have consistently demonstrated that the majority of recurrences occur within the first three years >> following initial diagnosis. In a population-based study by Zach et al., the overall recurrence rate was reported as 22.3% over a median follow-up of 64 months. Of these recurrences, 61% were local, 30% occurred in the groin, and 9% were distant, indicating the predominance of locoregional recurrence and the importance of effective post-treatment monitoring [59]. The role of ^18^F-FDG PET/CT in surveillance in vulvar cancer is not well established, and current evidence is limited. While data from other squamous cell malignancies suggest a potential role in early detection of recurrence and treatment response assessment, extrapolation should be interpreted cautiously.

Telemedicine-based psychosocial interventions may also complement long-term surveillance by reducing fear of cancer recurrence and supporting patient-centered survivorship care, although this remains outside the core imaging role of PET/CT and should be considered an adjunctive survivorship strategy [60].

PET/CT has increasingly been investigated as a tool for treatment response assessment and early detection of recurrence in gynecologic malignancies, for example, in breast cancer. Studies in this setting have demonstrated that response to therapy can be assessed through reductions or normalization of ^18^F-FDG uptake at the site of the original tumor following initiation of chemotherapy [61]. Although standardized response criteria have not yet been widely established, several studies suggest that reductions in SUVmax between approximately 55% and 65% after the second cycle of chemotherapy may indicate treatment response, whereas reductions less than 55% may be associated with poor response to subsequent therapy [62]. Supporting this finding, Kolesnikov-Gauthier et al. reported that a decline in SUVmax of less than 15% after the first chemotherapy cycle was strongly associated with treatment non-response [63]. Evidence from other squamous cell malignancies further supports the role of PET/CT in early recurrence detection. In patients with head and neck squamous cell carcinoma, FDG PET combined with high-resolution chest CT (HRCT) detected recurrent disease earlier and more frequently than HRCT or clinical examination alone. In that study, the median time to radiologic recurrence was six months, and PET/CT demonstrated superior diagnostic performance, with sensitivity, specificity, PPV, and NPV of 100%, 87.3%, 56.5%, and 100%, respectively, compared with 61.5%, 94.9%, 66.7%, and 93.8% for conventional imaging and clinical assessment [64].

Additional studies emphasized that PET-based metabolic parameters may outperform conventional cross-sectional imaging in predicting treatment response, tumor necrosis, and disease recurrence following radiation therapy [65].

While most metabolic response criteria derive from breast and head and neck SCC literature, direct vulvar-specific data remain scarce, and current practice extrapolates from these malignancies. Despite data specific to vulvar cancer being limited, emerging evidence suggests a similar potential benefit. In a study evaluating 63 patients with vulvar cancer undergoing restaging with ^18^F-FDG PET/CT, imaging findings were compared with histopathology and other imaging modalities. PET/CT demonstrated a sensitivity of 100%, specificity of 92%, PPV of 98%, and NPV of 100% for detecting recurrent disease. Moreover, PET/CT findings altered clinical management in 12 patients, shifting treatment from local therapy to systemic chemotherapy due to the detection of disseminated disease. Albano et al. reported that ^18^F-FDG PET/CT achieves good diagnostic performance in patients with suspected recurrent vulvar cancer, meaningfully influencing clinical decision-making, and further found that restaging metabolic findings correlated with prognosis [66]. These results are encouraging but should be interpreted within their acknowledged constraints: the evidence derives from a retrospective design, lacks histological confirmation of all PET/CT findings, and was drawn from a small cohort, which are limitations that reflect the broader difficulty of studying an uncommon imaging application in a rare disease. As the authors themselves emphasize, the definitive role of ^18^F-FDG PET/CT in recurrent vulvar cancer remains to be established through well-designed, multicenter prospective studies with histopathological correlation. We share this view and regard such studies, incorporating standardized SUV thresholds and direct comparison against MRI and clinical examination, as a key priority for advancing the field beyond the current, largely retrospective evidence base.

A further potential contribution of PET/CT is prognostic risk stratification, not merely lesion detection. Virarkar et al. recently evaluated imaging and clinical predictors in 45 patients with vulvar tumors and found that primary tumor SUVmax was associated with overall survival in univariate Cox analysis (HR 1.07 per unit increase; *p* = 0.008). Using an optimized cut point, SUVmax > 22.0 identified patients with substantially poorer survival, with a reported median survival of 1.92 years, alongside age and primary tumor size as adverse prognostic variables [67]. Although retrospective and not sufficient to define treatment thresholds, these findings support incorporating metabolic tumor burden into future prospective prognostic models and may help update the field beyond the diagnostic focus of earlier PET/CT reports.

## 9. Limitations of FDG PET/CT in Vulvar Cancer

Beyond the technical and biological factors discussed below, the overall quality of the evidence base warrants caution. Most vulvar-specific PET studies are small and single-center, predominantly retrospective, and enroll heterogeneous patient populations spanning primary staging, restaging, and recurrence. Diagnostic thresholds are not standardized: reported SUVmax cut-offs for classifying nodes as malignant vary substantially between studies, which limits the comparability of pooled sensitivity and specificity estimates and contributes to the wide range of reported values. These methodological constraints should be borne in mind when interpreting the performance figures summarized throughout this review.

### 9.1. Technical Limitations and Quantitative Variability

#### 9.1.1. The Partial Volume Effect (PVE)

As the spatial resolution of PET is typically limited to 4–6 mm, SUV parameters significantly underestimate the true metabolic activity of small primary tumors or micrometastases (often <5 mm in vulvar cancer). This “smearing” of the signal into adjacent tissues results in reduced SUVmax and SUVmean, potentially leading to false-negative findings in early-stage disease. Furthermore, automated thresholding is susceptible to spill-over artifacts from high-intensity physiological reservoirs [35].

#### 9.1.2. Inter-Site Variability

The absence of universal standardization (e.g., adherence to PERCIST criteria) across different clinical centers remains a barrier. This lack of uniformity limits the generalizability of quantitative metabolic data and complicates the comparison of SUV thresholds across multicenter cohorts or retrospective case series.

#### 9.1.3. Limited Specificity and Pitfalls in FDG PET/CT Interpretation

FDG uptake is not tumor-specific. Because FDG accumulates in any tissue with high glucose metabolism, benign or inflammatory conditions can produce uptake patterns indistinguishable from cancer. For example, normal physiologic processes in the pelvis (menstrual-cycle or menopausal changes) cause cyclic variations in endometrial and ovarian FDG uptake, which can confuse interpretation if not recognized. Inflammation and infection in the vulvar/vaginal area (e.g., vulvovaginitis, cellulitis, pelvic inflammatory disease) can show intense FDG activity, potentially masking coexistent tumor or falsely suggesting metastatic disease. Likewise, benign findings (e.g., endometriotic cysts, fibroids, or post-surgical granulation tissue) may take up FDG and mimic malignancy. Additionally, intense urinary bladder activity and patient motion may obscure subtle lesions in the vulvar region [20]. Conversely, some vulvar tumors—especially small, necrotic, mucinous, or low-grade lesions—may have low FDG avidity and yield false-negative scans. In short, both false positives and false negatives are possible, so PET/CT findings in the vulvar region must be correlated with clinical and anatomic imaging data [21,68].

### 9.2. Biological and Physiological Limitations

Although FDG PET has revolutionized cancer diagnosis and management, this modality carries several important limitations. SUV parameters are influenced by patient-specific variables, including blood glucose levels, body mass index (BMI), and the presence of inflammatory processes in the vulvar or inguinal region. Such factors may lead to false-positive metabolic activity that mimics or masks low-grade squamous cell carcinoma uptake.

A biological evaluation of PET findings needs to take into consideration the two main pathways of vulvar squamous cell carcinoma (VSCC) development. The first type of tumors, which is usually positive for p16, tends to occur at an earlier age, while the other one is often seen in older patients and arises in the context of inflammatory dermatoses, most commonly lichen sclerosus. HPV-negative tumors also tend to show a greater association with p53 abnormalities. Direct evidence, which would associate HPV/p16 positivity with the baseline FDG uptake, is scarce; therefore, the SUV is not a biomarker. However, HPV/p16 positivity might be important when evaluating the PET changes after therapy, because post-radiotherapy metabolic washout can represent not only the tumor biology but also sensitivity to the treatment [69]. Hormones, menopause, infections, wound healing, fibrosis, and inflammation induced by radiation can affect pelvic FDG accumulation and cause false-positive and -negative results. Involvement of the vulva in particular, due to urinary function, dermatitis, ulceration, previous surgeries, and fibrosis, can anatomically overlay with the area of the primary tumor bed. FAPI PET/CT can potentially evaluate the metabolism of the fibroblasts instead of tumor glycolysis and may be useful in cases of desmoplastic and fibrotic tumors. However, this imaging technique needs to be investigated further [70,71,72,73,74,75,76].

### 9.3. Post-Treatment Imaging Pitfalls

Therapy-induced changes can confound PET/CT. Surgical and radiation treatments distort normal anatomy and elicit FDG-avid inflammation and healing. Postoperative scarring, radiation fibrosis, or chronic inflammation (e.g., radiation proctitis or cystitis) can all cause abnormal FDG uptake in the treated field [77,78]. Complications such as fistula formation (e.g., vesicovaginal fistulas) are particularly problematic, as urinary FDG leaks into the wound or vulva and may be mistaken for tumor [79]. Delayed complications (radiation-induced fractures, osteoradionecrosis, chronic pelvic inflammatory changes) can appear months to years later and mimic tumor recurrence [80]. To avoid misinterpretation, PET/CT is generally deferred for several weeks after therapy: a common practice is to wait at least ~8–12 weeks after surgery or radiotherapy before scanning. This allows acute inflammation to subside and reduces false-positive findings [78]. Radiologists must be aware of each patient’s treatment history and expected post-therapy appearance to distinguish healing changes from residual or recurrent cancer.

### 9.4. Clinical Limitations and Cost Constraints

#### 9.4.1. Radiation Exposure

FDG PET/CT inherently involves ionizing radiation, as the patient is injected with a radioactive tracer and undergoes a CT scan, raising a theoretical carcinogenic risk [22]. Because of the associated radiation exposure, together with the relatively prolonged and complex acquisition protocol, PET/CT is selectively utilized, primarily in advanced-stage disease or when metastatic spread is suspected, rather than as a standard imaging modality for all cases of vulvar cancer. PET/CT imaging entails several preparatory and procedural steps, including a period of fasting, an approximate 60 min radiotracer uptake phase, and a scan duration of around 20–30 min, which may limit its feasibility for repeated use during follow-up. Although ^18^F-FDG PET/CT is widely available, the time-intensive nature of the procedure may still limit its practicality for frequent serial imaging in routine clinical settings. From a resource perspective, PET/CT carries a higher upfront acquisition cost than conventional imaging; this initial cost, however, should be weighed against its potential to refine staging, avoid unnecessary or more invasive procedures, and improve treatment selection, which may offset expenditure over the full care pathway. Formal cost-effectiveness data specific to vulvar cancer nonetheless remain lacking [23].

#### 9.4.2. Limited Sensitivity for Small Nodal Metastases

The PVE described above makes micrometastatic deposits (≤2 mm) essentially invisible on whole-body PET imaging. Large PET trials and meta-analyses underscore this gap. For example, a recent systematic review found a pooled sensitivity of only ~70% (per patient) for FDG PET/CT in groin nodal staging of vulvar cancer, meaning a substantial fraction of true metastases are missed [14]. PET/CT did show high specificity (often ≥90%), so a negative PET/CT scan may be reassuring, but a significant false-negative risk remains [24]. In contrast, surgical sentinel-node biopsy with thorough pathology (ultrastaging) detects tiny metastases that PET cannot. In the landmark GOG-173 study, immunohistochemical analysis of sentinel nodes identified tumors in 23% of cases that were negative on routine H&E staining, illustrating how often small deposits go undetected without detailed histology [25]. These micrometastases have real prognostic impact (GROINSS-V follow-up showed tailored treatment for small nodal disease can safely reduce morbidity) (ClinicalTrials.gov: NCT02969278; NCT05076942; NCT06476639; and NRG-GY024). Hence, PET/CT is useful as an adjunct (e.g., a positive FDG-avid node is meaningful, and a negative scan can add confidence), but it cannot obviate histologic lymph node assessment [24].

PVE, arising from the limited spatial resolution of PET/CT, represents a fundamental limitation and are the primary reason for reduced sensitivity in detecting subcentimetric nodal disease, particularly micrometastases (≤2 mm), thereby preventing FDG PET/CT from replacing sentinel lymph node biopsy in vulvar cancer staging. Typical PET scanners produce final image resolutions on the order of around 4–6 mm FWHM, and partial-volume averaging substantially reduces the measured signal from lesions smaller than around 2–3 times the system resolution.

On the other hand, PET/CT and sentinel lymph node biopsy (SLNB) serve different purposes. PET/CT is non-invasive, provides whole-body staging, allows visualization of disease outside the surgical field in the pelvis or even metastatic sites, and potentially gives indications about biopsy or radiation fields, although it is unable to provide sufficient spatial resolution or pathological ultrastaging to detect isolated tumor cells or micrometastases. SLNB is an invasive method that needs nuclear medicine mapping, surgical skills, and a pathologist. However, it directly examines the first lymph node basin and gives histopathological information useful for the selection of adjuvant therapy. Morbidity of the SLNB procedure is less than morbidity after completing inguinofemoral lymphadenectomy, although wound infections, development of lymphoceles, and lymphedema are still possible. Thus, negative PET/CT reduces the probability of macroscopic nodal disease, but does not give the same false-negative results that could be obtained with the help of successfully mapped and ultrastaged negative sentinel nodes. On the other hand, PET-positive nodes might give indications for biopsy or treatment field change, but cannot replace histopathological examination if its results determine further patient treatment. Cost-effectiveness analysis of PET/CT compared with SLNB in vulvar cancer has not been done yet [14,24,25,26,27].

## 10. Beyond FDG: Other Tracers in Vulvar Cancer

### 10.1. Fibroblast Activation Protein Inhibitor (FAPI)

Fibroblast activation protein inhibitor (FAPI) PET/CT represents a promising emerging imaging modality in gynecological malignancies based on its targeting of the tumor microenvironment rather than tumor-cell glucose metabolism [70,71]. Vulvar carcinomas, predominantly squamous cell carcinomas, exhibit a prominent desmoplastic stromal reaction rich in cancer-associated fibroblasts (CAFs), which overexpress fibroblast activation protein (FAP), a type II serine protease with minimal expression in normal tissues but marked upregulation in tumor stroma, fibrosis, and tissue remodeling. FAPI tracers specifically target this FAP overexpression within the tumor microenvironment [71].

Unlike FDG, which reflects tumor cell glucose metabolism, FAPI PET targets the tumor microenvironment by imaging FAP-expressing cancer-associated fibroblasts, enabling higher tumor-to-background contrast and improved lesion delineation. It demonstrates low physiologic uptake and is less affected by inflammatory activity, making it particularly useful in tumors with prominent stroma or low FDG avidity. However, its role in vulvar cancer remains investigational, with no dedicated clinical studies to date.

In a small retrospective series of gynecological tumors that did not include vulvar cancer, ^68^Ga-FAPI PET/CT showed high tracer uptake in primary and metastatic lesions and, in the subset undergoing both scans, higher tumor-to-background ratios than ^18^F-FDG [72]. A systematic review and meta-analysis likewise suggested that FAPI PET/CT may outperform FDG for detecting lesions in abdominal and pelvic malignancies, although none of this evidence is specific to vulvar cancer [73].

Direct evidence for FAPI in vulvar cancer is currently lacking. The ongoing FAPI_VULVA_1 trial (NCT06911801) is investigating FAP expression in vulvar squamous cell carcinoma specimens using ^18^F-FAPI-74, and its results are awaited to clarify any potential diagnostic role. The shared molecular target also raises theranostic possibilities for radioligand therapy in FAP-expressing tumors, which would be of interest in advanced or recurrent disease with limited treatment options [74,75]. However, no clinical evidence yet supports routine use in vulvar cancer.

### 10.2. Somatostatin Receptor–Targeted (SSRT) Imaging

Using agents such as ^68^Ga-DOTATOC and ^64^Cu-DOTATATE shows particular promise for identifying and staging rare neuroendocrine variants of gynecologic cancers, which may exhibit low FDG avidity but high receptor expression. This receptor-based approach not only enhances lesion detectability but also opens avenues for theranostic applications, including peptide receptor radionuclide therapy [16].

### 10.3. ^68^Ga-Pentixafor

This tracer targets CXCR4, a receptor involved in tumor growth and spread, representing an emerging approach. While this technique has shown promising results in hematologic malignancies, its application in solid tumors, including gynecologic cancers, remains limited and requires validation [81].

### 10.4. ^11^C-TYR (L-[1-11C]-Tyrosine)

This older tracer, studied in 1999, was investigated for detecting lymph node metastases by imaging amino acid metabolism. The research concluded it was not superior to physical palpation for this purpose, and it has not become part of standard clinical practice [82].

### 10.5. Targeted Nanobody-Based PET Theranostics

High-affinity targeted nanobodies represent another emerging theranostic platform for PET imaging and radioisotope therapy. Their small molecular size, rapid blood clearance, deep tissue penetration, and favorable tumor-to-background contrast may overcome some limitations of conventional antibody-based imaging [83]. In a recent preclinical and first-in-human evaluation, Zhang et al. developed CE-21, a high-affinity carcinoembryonic antigen-targeted nanobody that could be radiolabeled with 68Ga for PET/CT imaging or 177Lu for radionuclide therapy [83]. 68Ga-CE-21 demonstrated rapid clearance, low nonspecific uptake, and high-contrast imaging, while 177Lu-CE-21 showed dose-dependent tumor-growth inhibition in preclinical models [83]. Although this evidence is not specific to vulvar cancer, it illustrates the potential of nanobody-based PET theranostics in epithelial malignancies and may inform future tracer development if relevant molecular targets are validated in vulvar cancer.

## 11. Emerging Advances and Future Directions in PET Imaging Technologies

PET detector technology has undergone major advances to achieve higher sensitivity, including the development of new detector materials such as lutetium-based crystals like lutetium yttrium orthosilicate (LYSO), which have enhanced light output and timing performance [84]. Moreover, the introduction of silicon photomultipliers (SiPMs) and digital photon counters (DPCs) has improved light collection efficiency and overall sensitivity of PET detectors [85]. Time-of-flight (TOF) technology is also a major advance in PET imaging, improving localization by measuring the time difference between photon emission and detection. This allows for a higher accuracy in the positioning of annihilation events, resulting in better spatial resolution. Advances in detector materials, electronics, and reconstruction algorithms now enable TOF PET to produce images with markedly higher clarity [86]. Despite these theoretical advantages, the clinical impact of modern PET detector technology and TOF imaging has not been specifically validated in vulvar cancer [87,88,89,90,91,92].

Beyond hardware-based advances in PET detector sensitivity and time-of-flight reconstruction, image-domain super-resolution algorithms may help mitigate the partial volume effect (PVE), a key limitation in detecting small primary lesions, micrometastatic nodal deposits, and low-volume recurrent disease in vulvar cancer. A PET-specific convolutional neural network method developed by Song et al. combined low-resolution PET with spatial information and, where available, high-resolution MRI; it improved edge and contrast recovery in simulation and clinical neuroimaging datasets [93]. DDNet is a dynamic lightweight super-resolution framework for arbitrary scaling factors [94], but it is a general image-domain method and has not been validated for PET or vulvar cancer. These approaches may improve visualization of subtle metabolic signals currently underestimated by standard PET resolution, but their use in vulvar cancer remains theoretical and requires prospective validation [93,94,95].

Modern computational methods and Artificial Intelligence (AI) have reshaped PET image processing, improving reconstruction, noise suppression, motion handling, and lesion outlining. These techniques sharpen diagnostic accuracy while allowing shorter scans and lower radiation doses, making examinations safer and more efficient. They also speed up radiotracer development and enable more individualized dosimetry, reinforcing PET’s value in theranostic applications [84].

Graph neural networks (GNNs) may provide a complementary artificial-intelligence approach for multimodal oncological imaging by extending deep learning to non-Euclidean data structures. In medical imaging, graphs can represent pixels, patches, anatomical regions, lesions, or multimodal features as nodes, while edges encode spatial, structural, or functional relationships. This framework has been applied to segmentation, classification, registration, reconstruction, and multimodal fusion; however, its use in vulvar cancer imaging remains unvalidated and should currently be considered investigational [96]. Integrated GNN architectures may combine graph convolutional networks (GCNs), graph attention networks (GATs), and graph sampling aggregation networks (GraphSAGE) through cascaded, parallel, or feature-level fusion strategies. Reference [97] specifically reviews all three branches and their integration pathways, including scalability and adaptive feature weighting.

However, evidence on AI use in PET imaging for vulvar cancer remains very limited. To date, only a single vulvar cancer-specific study has applied radiomic analysis to ^18^F-FDG PET/CT images of the primary tumor [98]. The authors concluded that PET-based radiomics in vulvar cancer is feasible and may offer additional prognostic insight beyond standard pathology, while stressing that larger studies are required before such AI-derived markers can be used in routine practice. This represents a first step toward AI-driven quantitative imaging in this context. Other AI and PET radiomics research in gynecologic oncology has focused on cervical, endometrial, and ovarian cancers, with vulvar cancer typically noted only as an emerging area with scant supporting data [95,99,100].

Evidence from other disease models suggests that multimodal AI systems integrating imaging-derived features with clinical and serum biomarkers can improve staging and progression-risk stratification; similar PET/MRI-clinical fusion approaches warrant prospective evaluation in vulvar cancer [101].

There are few prospective trials available. There is only one published prospective trial of PET-based radiotherapy in the vulva reported in this literature review, namely the PET/CT-based adaptive radiotherapy trial by Shenker et al. [42]. In terms of emerging tracers, the single prospective registered trial for vulva, FAPI_VULVA_1 (NCT06911801), represents an in vitro feasibility trial on the binding of ^18^F-FAPI-74 in stored vulvar squamous cell carcinoma biopsies, and not a whole-body imaging clinical trial [76]. In terms of PET/MRI and PET radiomics, the available evidence for the vulva is limited to small studies or retrospective ones, such as the first PET/CT-based radiomics trial [98]; there are no large prospective studies available specifically for the vulva. Thus, Table 3 describes the current trial landscape.

**Figure 3:**
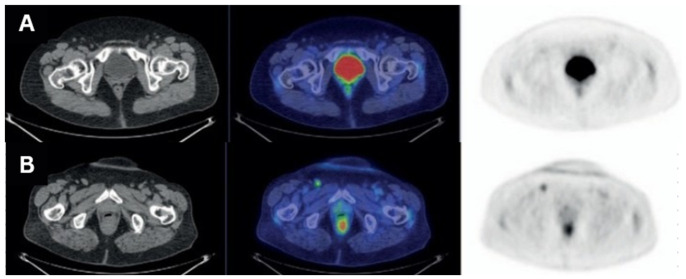
**Panel A**: Representative images from a false-negative case of vulvar cancer patient. Axial CT, fused, and PET images (from left to right) demonstrate no pathological uptake of FDG in the bilateral groin. Histopathological analysis subsequently revealed partial metastatic involvement of a left inguinofemoral lymph node. **Panel B**: Another patient with false-positive findings. Axial CT, fused, and PET images (from left to right) demonstrate increased FDG uptake in the right inguinal region (SUVmax 3.53), with no evidence of metastatic disease on histological examination. Adapted under CC BY 4.0 from [56].

In summary, ^18^F-FDG PET/CT represents a valuable adjunct imaging modality in the management of vulvar cancer, particularly in locally advanced disease and in the evaluation of nodal and distant metastases (Figure 3). Its high specificity and negative predictive value support its role in risk stratification and treatment planning; however, limited sensitivity for micrometastases precludes its use as a standalone staging tool. Integration of PET findings within a multimodality imaging framework, alongside histopathologic evaluation, remains essential. Future directions should focus on standardization of quantitative PET metrics, validation of volumetric and radiomics-based biomarkers, and prospective studies to better define its role in the personalized management of vulvar cancer.

## 12. Conclusions

In conclusion, FDG PET/CT is a valuable adjunct in the evaluation of vulvar cancer, especially in patients with locally advanced disease, suspected nodal involvement, or concern for distant metastasis or recurrence. Its whole-body functional information can improve staging, support treatment planning, and help guide radiotherapy, but it should not be used in isolation because of its limited sensitivity for micrometastases and its susceptibility to false-positive findings from inflammation or post-treatment changes. MRI remains superior for local tumor assessment, and sentinel lymph node biopsy continues to be essential for groin staging in appropriate patients. False-positive inflammatory or post-treatment uptake may generate anxiety, additional imaging or biopsy, unnecessary surgery, or inappropriate expansion of radiotherapy fields; therefore, management-changing positive findings require anatomical correlation, multidisciplinary review, and tissue confirmation when feasible [20,21,77,78,79,80]. The greatest technical benefit is expected from modern digital PET/CT systems that combine silicon photomultipliers, time-of-flight (TOF) reconstruction, point-spread-function or other high-resolution reconstruction, rigorous quality assurance, and complementary MRI or diagnostic CT. TOF improves event localization, signal-to-noise ratio, and lesion conspicuity, but it does not overcome the histologic limit for micrometastases, and no minimum system resolution threshold has been validated specifically for vulvar cancer [84,85,86,87,88,89,90]. Overall, the current evidence supports a complementary, multimodality approach rather than the replacement of established diagnostic methods. Future research should focus on larger prospective studies, standardized interpretation criteria, and emerging tools such as new tracers and artificial intelligence-based analysis to improve diagnostic accuracy and clinical decision-making. These advances may help refine staging, reduce unnecessary invasive procedures, and support more personalized care for patients with vulvar cancer.

## Figures and Tables

**Table 1:** FIGO 2021 staging for vulvar cancer [9].

Stage	Description
I	Tumor confined to the vulva IA: Tumor size ≤ 2 cm and stromal invasion ≤ 1 mm ^a^ IB: Tumor size > 2 cm and stromal invasion > 1 mm ^a^
II	Tumor of any size extending to adjacent perineal structures (lower third of urethra, lower third of vagina, or anus) with no regional lymph node metastasis
III	Tumor involving adjacent upper perineal structures and/or regional lymph node metastasis (non-fixed, non-ulcerated) IIIA: Extension to upper two-thirds of urethra or vagina, or involvement of bladder/rectal mucosa, and/or regional lymph node metastases ≤ 5 mm IIIB: Regional ^b^ lymph node metastases > 5 mm in size IIIC: Regional ^b^ lymph node metastases with extracapsular spread
IV	Advanced disease with fixation to bone, fixed or ulcerated lymph nodes, or distant metastasis IVA: Tumor fixed to pelvic bone or presence of fixed/ulcerated regional lymph node metastases IVB: Presence of distant metastases

^a^ Depth of invasion is measured from the basement membrane of the deepest, adjacent, dysplastic, tumor-free rete ridge (or nearest dysplastic rete peg) to the deepest point of invasion. ^b^ Regional refers to inguinal and femoral lymph nodes.

**Table 2:** Summary of studies that assess the role of CT CAP and PET/CT in lymph node metastases in vulvar cancer.

Imaging Modality	Study	Sensitivity	Specificity	PPV	NPV
CT-CAP	Andersen et al. ^a^ [48] Land et al. ^b^ [49] Bohlin et al. ^b^ [50] Pounds et al. ^a^ [51]	60% 58% 43% 59%	90% 75% 96% 78%	38% 58% 88% 62%	96% 75% 73% 76%
PET/CT	Triumbari et al. ^c^ [14] Cohn et al. ^a^ [18] Collarino et al. ^a^ [36] Piero et al. ^b^ [43] Lin et al. ^a^ [52] Kamran et al. ^b^ [53] Dolanby et al. ^a^ [54] Crivellaro et al. ^b^ [56] Garganese et al. ^a^ [57] Oldan et al. ^b^ [58] Rufini et al. ^b^ [24]	70% 80% 95% 100% 92% 50% 100% 50% 56% 100% 73–86%	90% 90% 78% – 91% 100% 100% 67% 88% 89% 66–85%	86% 80% 69% – 85% 100% – 58% 38% – 52–68%	77% 90% 96% – 95% 57% – 59% 93% – 88–91%

^a^ Prospective study. ^b^ Retrospective study. ^c^ Systemic review and meta-analysis.

**Table 3:** Prospective and active PET-related evidence in vulvar cancer.

Area	Direct Vulvar-Specific Evidence and Current Status
FDG PET/CT adaptive radiotherapy	Published prospective institutional study by Shenker et al. in locally advanced vulvar cancer; supports feasibility and dosimetric benefit but requires multicenter validation [42].
FAPI PET	FAPI_VULVA_1/NCT06911801 is an observational in vitro study of ^18^F-FAPI-74/FAP expression in vulvar squamous carcinoma specimens; it is not a clinical whole-body staging trial [76].
PET/MRI	No dedicated prospective vulvar-specific PET/MRI validation trial was identified; current support is indirect from related gynecologic malignancies.
PET radiomics/AI	Current vulvar-specific evidence consists of a retrospective ^18^F-FDG PET/CT radiomics experience; external validation and outcome-linked prospective cohorts are needed [98].
PET/SLNB-integrated staging	No prospective workflow trial has established PET-directed biopsy/SLNB integration; future studies should assess false-negative outcomes, patient burden, and cost-effectiveness [14,24,25,26,27].

## Data Availability

The original data presented in this literature review are openly available in the published literature and indexed databases, including PubMed and Scopus through the references cited in this review.
